# African Herbal Medicines: Adverse Effects and Cytotoxic Potentials with Different Therapeutic Applications

**DOI:** 10.3390/ijerph18115988

**Published:** 2021-06-02

**Authors:** Kunle Okaiyeto, Oluwafemi O. Oguntibeju

**Affiliations:** Phytomedicine and Phytochemistry Group, Oxidative Stress Research Centre, Department of Biomedical Sciences, Faculty of Health and Wellness Sciences, Cape Peninsula University of Technology, Bellville 7535, South Africa; okaiyetok@cput.ac.za

**Keywords:** African herbal medicines, adverse effects, toxicity, therapeutic values

## Abstract

The African continent is naturally endowed with various plant species with nutritional and medicinal benefits. About 80% of the people in developing countries rely on folk medicines to treat different diseases because of indigenous knowledge, availability, and cost-effectiveness. Extensive research studies have been conducted on the medicinal uses of African plants, however, the therapeutic potentials of some of these plants has remained unexploited. Over the years, several studies have revealed that some of these African floras are promising candidates for the development of novel drugs. Despite the plethora of studies on medicinal plant research in Africa, there is still little scientific data supporting the folkloric claims of these plants. Besides, safety in the use of folk medicines has been a major public health concern over the year. Therefore, it has become mandatory that relevant authority should take measures in safeguarding the populace on the use of herbal mixtures. Thus, the present review extracted relevant information from different scientific databases and highlighted some problems associated with folk medicines, adverse effects on reproductive systems, issue about safety due to the toxicity of some plants and their toxicity effects with potential therapeutic benefits are discussed.

## 1. Introduction

The African continent is magnificently endowed with different plant diversity, mainly due of the prevailing climatic conditions and this advantage has supported the richness of secondary metabolites in the plants for surviving under harsh environmental conditions [1,2]. Besides, the ultraviolet rays in this region are stronger than those observed in other parts of the world, and this also enables the plants to accumulate some important bioactive substances of economic importance [1]. These reasons justify the exploitation of these plants for their therapeutic values that have been in continual use in this region for the past several decades [3,4].

According to a report by the WHO [5], about 80% of the people in developing countries rely on traditional herbal mixtures to treat different diseases. Most villages in Africa still depend solely on traditional herbal mixtures as a source of health treatments because of their beliefs and culturally acceptable indigenous knowledge, accessibility, and affordability. Many herbal mixtures are indubitably expedient for maintaining good health or treating diverse diseases [6,7]. Besides, numerous African rural dwellers believe that since their ancestors used herbal mixtures/concoctions for their wellbeing in the past and with no side effect, they habitually assume that because the herbal mixtures are natural, therefore their safety is guaranteed. Unfortunately, this assumption has led to several instances of organ damage and death of the users [8,9]. In addition, traditional healers are very secretive about their indigenous practices handed down from their ancestors to the new generation and this makes their treatment prescriptions vague, often resulting in overdoses of the mixtures by their patients since no regulatory body controls the usage of herbal medicines. Perhaps nothing indicates to the patients that “too much of a good thing” could be dangerous. Sadly, some people ignorantly combine herbal mixtures with orthodox medicines without a doctor’s prescription and perhaps, not considering their adverse interactions. It is important to note that uncontrolled consumption of herbal mixtures could lead to liver damage, kidney failure and stomach upsets, diarrhoea, etc. [10]. Herbal mixtures contain some bioactive compounds that are potentially toxic. The reports documented in the literature have shown that despite the crucial role of herbal medicine for man, some plant species are reported to cytotoxic at high dosage. This simply means that a very safe compound can be toxic at a high dose and vice versa. The toxicity-related issues about herbal medicines such as carcinogenicity, hepatotoxicity, mutagenicity, genotoxicity have been highlighted by Fanell et al. [11]. Therefore, safety relating to herbal mixtures or products cannot be disregarded, as knowledge is key in preventing overdoses or abuse. On the other hand, deforestation of the natural habitat due to anthropogenic activities has mounted pressure on the African ecosystem [1]. Nevertheless, it is worth noting that Africa represents about 25% of the world trade in biodiversity and it is still surprising that despite the contribution, only a few drugs from African plants have been commercialized compared to other continents.

## 2. Factors Influencing the Use of Herbal Medicines

According to the WHO [5], traditional medicine is the total of knowledge, skills, and experiences based on the theories, beliefs, and practices indigenous to different cultures, whether explicable, that are used to maintain health, and to prevent, diagnose, improve, or treat physical and mental illnesses. Hence, these practices have been explored by people since time immemorial to treat various diseases before the arrival of orthodox medicines. These herbal medicines may include herbs, herbal materials, herbal concoctions and finished herbal products [3]. Over the years, the patronage of people towards herbal medicine has been increasing tremendously because of several reasons that include but are not limited to the following:

(a) The belief of rural dwellers and their indigenous cultures: Numerous rural dwellers have strong convictions that their beliefs and cultural practices for treating diseases are far better than modern medicine. Some even have a strong phobia towards orthodox medicines. As a result, nothing can make them opt for orthodox medicine even if the government is willing to pay their medical bills. They prefer consulting their gods and searching for a practical solution by sacrificing animals to please their gods. Different African traditional healers have their unique ways of consulting their oracles and communicating their findings to clients on their health-related issues and the possible solutions. In addition, these patients are more comfortable in expressing their health problems to the traditional healers in their local dialects than to a professional medical doctor [12].
(b) A perception that herbal medicines are natural and safe: Most herbal medicine users believe that herbal medicines are safe because they are products of plant naturally found in the environment and thus, they assume that being natural implies safety with no adverse effect compared to those reported from the use of orthodox medicine [13].
(c) Accessibility and cost-effectiveness: Herbal medicines have been the only option source of treatment in some African communities because they are cheap and the raw materials for preparing the mixtures are easily available. Besides, their cost-effectiveness cannot be compared with orthodox medicines, as most people living in these areas are poor and unable to afford the cost of modern treatments. These salient reasons have mitigated the rural dwellers towards the use of herbal medicines [14].
(d) Superior efficacy of herbal medicines: Many people believe that herbal medicines are more potent than orthodox medicines and the failure of orthodox medicines to treat some complicated health problems has diverted people’s attention in seeking herbal mixtures as an alternative source. Herbal mixtures contain several bioactive compounds that are believed to work together in synergy and thus enhancing their potency over orthodox medicines [15,16].
(e) Confidentiality of health problem information: Most people are not comfortable when information regarding their health problem is revealed to many people other than their doctor [13]. All patients have files containing their health information and the medical record clerks are responsible for handling these files. However, local people who use herbal medicines feel safer and prefer to discuss their health problem with the traditional healers to a modern health system where their files will be handle by different people on duty.
(f) Self-medication: This habit is common among the people living in the rural and semi-urban communities in African countries since the plant materials are easily accessible coupled with the little knowledge they acquired from generation about herbal concoctions, they tend to prescribe herbs preparations for people around them with similar kind of illnesses. The assumption is that since the concoctions have worked for other people, they would also serve the same purpose for any sick person with related symptoms [6].
(g) Fear of erroneous diagnosis: Most people prefer to opt for traditional medicines because of the fear of the wrong diagnosis from the modern health system. There are several reasons why a patient can be wrongly diagnosed in the hospital and such factors include unskilled medical operators (lack of advanced training of medical operators on the usage of new medical equipment), failure of old medical equipment, over the labour of medical staff, underpayment of medical staff, the inexperience of medical staff, mismanagement of patient’s file, inconsistency/changing of the medical doctor in charge of a patient [13].
(h) Long waiting period and queue involved to see a medical doctor: The queue involved, and time spent in seeing a medical doctor at the hospital is unbearable for most people in Africa, most especially, people without medical aid that patronize the government hospitals. Most hospitals in Africa are understaffed, so the population of patients overwhelms the capacity of these hospitals, and consequently, patients often seek an immediate alternative to solve their health problems. In some cases, sick people in the village might need urgent attention, with no ambulance available in the villages to convey them to the modern hospital in the town or city. In addition, a bad road network in the villages has been a huge challenge for the rural dwellers to get proper medical treatment. Besides, even the so-called mobile clinics are always frustrated or unwilling to go to the villages to attend to the poor people because of the poor road network, consequently, the villagers tend to opt for the most available alternative treatment [17].
(i) Advertisement of herbal products: Over the years, the market strategies of herbal mixtures or products in different ways have captured people’s attention in testing the efficacy of these products. Some traditional healers go on the street advertising their products by sharing handbills, pasting their posters everywhere in the town, or even employing marketing agents that will help them display their products on the television, different social media platforms. Marketing promotes business because of the awareness it creates for people and the visibility of a product is usually enhanced with an advertisement [18].

## 3. WHO Views on African Herbal Medicine

WHO has a substantial contribution to the acknowledgement and acceptance of traditional herbal medicine in African countries. Between 2002–2005, WHO provided a framework to promote traditional medicine to reduce the high death rate occurring in developing countries. WHO inspires African member states to endorse and incorporate folk medicine in their health system [19]. Therefore, four strategic objectives to incorporate folk medicine into the national healthcare systems include [20,21]:

i. To ensure that traditional medicines are easily accessible, available, and affordable for the poor populace.
ii. To ensure that traditional medicines are of high quality and effective without compromising on their safety for the populace.
iii. To promote the therapeutic use of traditional medicines for consumers.
iv. To ensure that traditional healers/specialists get the appropriate training and education required to improve on the preparation and prescription of their herbal products.

## 4. Justifications for Research on African Medicinal Plants

A plethora of supporting documents in the literature have validated the efficacy of African medicinal plants for the treatment of different human illnesses, however, there are still several gaps that are needed to be addressed, especially concerning the safety of these herbal mixtures or products to public health on the African continent. Thus far, the extensive research on African plants is geared towards drug discovery and development; there is a dire need to compile a list of promising African flora with significant therapeutic values, as most of these plants are not well documented. Likewise, there is a need to introduce African traditional healers to modern scientific practices. For the acceptance of folk medicines, the issue relating to active principles in the plant extracts, identification of secondary metabolites, mechanism of actions, and their toxicology need to be addressed. For that reason, WHO instigates further rigorous research on traditional medicines to substantiate their potency and establish safety for their usage [19].

### Acceptance of Traditional Medicines by Developed Countries

Folkloric medicine has been embraced by other people beyond their original indigenous culture, and this is referred to as complementary or alternative medicine [11,19]. The utilization of traditional medicines has been accepted in several advanced nations of the world, and complementary or alternative medicine has gained huge popularity in Europe, North America, and Australia [22,23]. The people living in these regions use medicinal herbal products to promote healthier living, as a blood cleanser, diet supplements, or for reducing body size. This therapeutic option drastically reduces the amount of money spent to purchase expensive drugs at the pharmacy and justifies the growth in the marketability of herbal products in these regions [6,24].

## 5. Safety of Traditional Herbal Medicines

Despite the numerous advantages associated with folkloric medicines, the big question is always about their safety to public health. The primitive techniques used by traditional healers are not modern or either scientific, hence, there are high chances of microbes and heavy metals contamination in the herbal products that could affect the health of the populace [16]. Therefore, the relevant authority needs to give a directive on how to protect the public from the negative effects (including death) that could result from the use or abuse of herbal products [25,26,27]. Microbial contamination is often common in herbal medicines, and it is always difficult for traditional healers to prevent or control this contamination. Thus, there is a need to recommend some quality control guidelines in their practices and perhaps awareness regarding the potential health risks associated with the use or abuse of herbal mixtures [25,27].

### 5.1. Adverse Effects of African Medicinal Plants and Role in Humans’ Reproductive System

In African communities, the introduction of herbal mixtures to the market is a norm even without scientific evaluation to ascertain their safety. There is also a belief that herbal products are safe. Besides, traditional healers lack regulatory control to guide their products, all these factors have worked together to misinform millions of people, resulting in the death of people [16]. Apart from microbial contamination, heavy metals have been reported to be contributors to the toxicity of herbal products that have led to life-threatening situations or death [6]. Several plants contain toxic bioactive compounds that can disturb normal physiological activities by chelating with cellular macromolecules such as DNA and proteins, leading to cellular toxicity and mutation [28]. The problem of herbal toxicity could result from production factors such as incorrect use of plants, species or plant parts, the quality of herbal products, high level of impurities, contaminants, and adulterants. Other possible factors could be resulting from the patient, such as co-morbidity, co-medication, and self-medication [29]. Several studies in the literature have highlighted the adverse effect of medicinal plants on the liver [30,31]. Liver enzymes are key biomarkers that indicate the level of liver damage and their presence in the blood is used to monitor liver disease in patients in the developing countries of the world [32]. In addition, the clinical symptoms of liver damage include asymptomatic mild biochemical irregularities to severe hepatitis with jaundice [33]. In agriculture, organophosphate pesticides are usually used to control pest in developing countries [33]. For instance, dichlorvos acts by irreversibly inhibiting acetylcholinesterase enzyme (AChE) at the cholinergic junctions of the nervous system and produces hepatotoxicity in rats [34]. Furthermore, aflatoxin, a potent hepatocarcinogen and hepatotoxin, is another potential microbial contaminant that has been identified in herbal products [35].

There is a general notion that traditional healing is dominated by men, as the percentage of male traditional healers is far greater than that of females. Notwithstanding this, the rich knowledge of indigenous women about folk medicines cannot be underrated [36]. Besides, female patients are always comfortable discussing women’s problems with the female traditional healers. Medicinal plant intake has contributed substantially to treating women-related health problems such as irregular menstruation, birth control, fertility, abdominal pain, pregnancy, waist pain, vomiting during pregnancy, child delivery, and postpartum care, lactation, infant care, etc. [37,38,39]. Examples of the medicinal plants commonly used by pregnant women includes *Zingiber officinale*, *Allium sativum*, *Ocimuml amiifolium*, *Eucalyptus* and *Rutachalepensis*, etc. [40]. For example, the usage of *Clivia miniata* has been reported to be associated with some side effects such as salivation and diarrhoea while *Callilepis laureola* causes confusion, convulsion, hepatic, and renal failures. Also, *Scadoxus puniceus* has been reported to be associated with visual disturbances and dizziness [41].

Medicinal plants have been folklorically used for the treatment of different types of reproductive diseases. However, contaminants such as heavy metals and persistent organic pollutants picked up by medicinal plants from the environment, especially when their concentrations are above the permissible limits, disrupt the normal physiological function of the human reproductive system [42,43]. Contaminant toxicity in plant extracts has been reported to be associated with a decrease in sex organs, sex performance, implantation rate, fertility, chromosome aberration, and mitotic inhibition in some in vivo studies [44,45,46]. These contaminants in plant extracts could trigger the production of free radicals and higher accumulation may lead to oxidative damage of the organ or tissues and macromolecules followed by oxidative stress thereby resulting in impaired reproductive systems [47,48,49]. For example, the report of a study conducted in Nigeria by Nwangwa [50] on ethanolic extract of *Xylopia aethiopica* showed a decrease in sperm viability, motility, and counts in rats. Another study by Akbarsha et al. [51] with *Andrograpis paniculata* in rats caused a decrease in spermatogenesis and degenerative changes in seminiferous tubules.

It is quite unfortunate that folk medicines are the only source of treatment for pregnant women in some Africa countries [52,53]. Normally, conventional antimalarial drugs are encouraged for pregnant women [4], however, the cost implication of these drugs in the developing countries by low-income earners has impelled this category of people to seek folk medicines, without considering the side effects on their foetus [54]. In addition, the use of medicinal plants for child delivery is a normal practice in some rural areas in African countries [55].

### 5.2. Hurdles to Safeguarding the Quality/Safety of Herbal Products

With the drastic upsurge in the number of people interested in herbal products, adverse effects associated with the use of these herbal products have posed a serious threat to public health. Hence, there is a dire need to monitor the production of these products, enlighten the populace about their toxicity and give adequate advice on their consumption and use [56,57]. Several challenges affect the quality of herbal products, which could result from the following:

i. Insufficient knowledge or information about the plant species: Most people that practice traditional medicine in Africa do not have good knowledge about the plants used in the treatment of different ailments since they strongly believe in the information passed to them from one generation to another. They do not know the scientific information about the toxicity of one plant to another when used in combination. Besides, the collection or harvest time for medicinal plants is one of the significant factors that affect the potency of the plant for their therapeutic usages and when the traditional healers lack the basic information about the plant, there is a high possibility of misidentification or the use wrong plant species that could result in the toxicity of the herbal mixtures or products [13].
ii. Lack of quality control on the herbal products: The preparation of most herbal products in the market or those used by the people in the villages are devoid of quality control assessment and the chances of their toxicity or adverse effect on human are very high, though some have been proven to be promising. There is no regulatory or standard set aside for the preparation of herbal products in the villages in Africa, hence, microbial contamination from the harvesting stage, production, and packaging is common in these products. Hence, the quality of materials used for the preparation of the herbal products determines the level of their safety [58].
iii. Lack of standard preparation methods: Different communities in Africa have their ways of preparing herbal mixtures and the method used in the preparation influences the extraction of the bioactive compounds in these plants, which are significant to their therapeutic values. Lack of standard methods may indirectly affect the difference in the efficacy of plants from various communities [59].
iv. Complex nature of the plant extracts: Several researchers have reported that the plant extracts contain many bioactive compounds and the complex nature of the method used in isolating and purifying individual bioactive compounds is critical and such analysis might not be possible in a local setting [60].
v. Overdose prescription: The prescription of herbal mixtures/products by traditional healers in the African communities is one of the factors that have resulted in many deaths in this region [61]. Most herbal mixtures contain several bioactive ingredients and adequate knowledge about the pharmacokinetics and the mechanism of actions of these products is lacking, hence, the prescription about the dosage used by the patients is not evidenced-based, which consequently leads to liver or kidney damage [8].
vi. Lack of scientific proof: Most herbal products in African countries lack any scientific validation [62].

## 6. Cytotoxic Effects of African Medicinal Preparations with Different Therapeutic Uses

From ancient times, the plant kingdom has been a reliable source of therapeutic agents for the treatment of different human illnesses because of the secondary plant metabolites [63]. Usually, plants produce numerous secondary metabolites as a defense mechanism against an array of pathogens [64]. The abundance and concentration of these secondary metabolites differ from plant to plant, geographical locations as well as abiotic factors that trigger their production [65]. A report by Samuelsson [66] has revealed that about 25–28% of orthodox drugs originated from plants. As a result, it has been predicted that the plant-based drug market could grow up to US$ 39.2 billion in 2022 [67]. Thus, continual screening of African biodiversity to search for cost-effective, novel, and effective plant secondary metabolites could play an important role in the biopharmaceutical industries [68,69,70]. However, it is worth noting that from a biological perceptive, the cytotoxicity of a plant does not only mean adverse effects, as this also has some therapeutic uses, and these have been well documented in the literature [71]. Unlike some protective roles such as antioxidant, antidiabetic, hepatoprotective, neuroprotective activities demonstrated by African plants, their cytotoxic effects have been reported to be beneficial to humans [72,73].

### 6.1. Antibacterial Activity

Bacterial infections represent a high percentage of the global causes of ill health and mortality because of the high prevalence of multidrug-resistant bacteria leading to antibiotic treatment failure thereby exposing the public to risk therefore, immediate intervention is highly imperative [74,75,76]. On the other hand, plants represent a significant source of novel antimicrobial agents. Plants are always exposed to different stresses from abiotic and microorganisms’ assault, and this prompts them to produce some defensive substances with antimicrobial activity [77]. Remarkably, these secondary metabolites produced by various plants have been recognized to have some therapeutic uses, including antibacterial activity [78,79]. A perusal of the literature indicated that several antibacterial studies on African plants have been documented [63,80,81]. However, poor toxicological documentation of plants used in folk medicines is the major limitation facing Africa traditional medicine, and this research gap has been the subject of active research lately [82]. Table 1 presents some African plants that have been reported to have antibacterial activity.

### 6.2. Antiplasmodial Activity

Malaria remains one of the most prevalent public health issues in the African countries of the world [94,95]. The high resistance of malaria parasites to the existing antimalarial drugs poses a serious threat to public health globally [96]. Hence, there is a pressing need to strategize on how to identify effective, inexpensive, and innocuous novel antimalarial agents. As part of this perpetual quest, African researchers and foreign collaborators have explored African biodiversity in the exploration of secondary metabolites from extracts of African plants for new antimalarial agents [94]. Traditionally, herbs have been the major treatment of malaria in most developing countries, especially in African countries [97]. These plants have been reported to be promising and could serve as a good alternative source for the isolation of lead compounds that could be used in the development of new antimalarial agents from this continent, and this could solve the problem of resistance of the malarial parasites [98]. Nevertheless, further studies on their toxicities such as carcinogenicity, teratogenicity, mutagenicity, toxicity should be the subject of active research to ensure their safety. Table 2 summarizes a few African florae with proven antiplasmodial activity.

### 6.3. Antifungal Activity

Fungal infections have been recognized as one of the deadly diseases that are difficult to treat in humans, animals, and plants because of the multidrug-resistant of the fungal pathogens to the existing antifungal agents. Besides, the current antifungal drugs have adverse effects both on the patients and the environment [121,122]. Thus, the need to search for alternative cost-effective and effective alternatives is indispensable. On the other hand, rural dwellers are highly knowledgeable about the culture and traditional practices used by their ancestors for treating fungal infections [123,124]. Herbs have been the only source for the treatment of fungal diseases and as a result, the antifungal activity of these plants has been validated by some African researchers to establish the therapeutic claims of these plants. Hence, continual ethnobotanical survey and screening of African plants in search of novel bioactive compounds that could be used in the development of effective antifungal drugs have been the focus of research in this field [125,126]. Table 3 summarises a few African florae with proven antifungal activity.

### 6.4. Anticancer Activity

Cancer is one of the topmost killer diseases globally, and it is predicted to be a major cause of death in the future [140]. It is a global public health issue affecting both developed and developing countries of the world, despite the innovation in the different therapy treatments [141]. This disease is a distressing issue, especially when not detected at the early stage, and the treatment costs are too high for the poor to afford [142]. However, radiotherapy and surgery have been acknowledged to be some of the most successful treatments, but the side effects and high treatment cost cannot be neglected [143]. Thus, this necessitates the urgent need to search for alternatives. Over the years, the use of herbal products for the treatment of different human diseases such as cancer has gained wide attention [144,145]. This means that plants or herbs may provide the main active principles for the development of new anticancer agents [146,147]. As a result, extensive studies on the anticancer activity of African plants have been well reported in the literature [148,149,150]. It has been established that bioactive compounds produced for plants’ defense have the potential to inhibit cancer cell growth. In addition, the report of Kawashima et al. [151] has highlighted that some of these bioactive compounds are toxic to humans. Regrettably, the toxicity of these plants has been documented in many cases to originate from various contaminants, such as pesticides, heavy metals, and other pollutants in the environment [29]. Evidence to validate the therapeutic values of these plants is still scarce, hence, there is a need for further research to confirm the traditional anticancer claims of these plants [152,153,154]. Several researchers have been investigating how the antioxidant potential of a plant can be related to its anticancer effects [155]. The bioactive compounds responsible for the antioxidant properties could however be different from those accounting for the antiproliferative effect. Nevertheless, the most important approach should be directed towards producing anticancer agents that could completely inhibit the growth of cancerous cells with no effect on normal cells. However, the findings of Lobo et al. [156] have highlighted that oxidative stress caused by the excessive reactive oxygen species such as superoxide anion (O_2_^•–^), nitric oxide (NO), hydroxyl radical (^•^OH) and peroxy radicals (ROO^•^) are involved in the pathogenesis of several pathologies, including cancer. The reactive species tend to cause DNA mutations, which could play a significant role in cancer [140]. Therefore, using plant-based antioxidant agents such as flavonoids, alkaloids and polyphenols could reduce the uncontrollable cell division caused by oxidative stress imposed by free radicals [157]. Thus, the use of natural antioxidants could play a lead role in the development of effective anticancer agents [158,159]. It is also worth noting that natural compounds have multi-target cellular effects [72,160,161]. Hence, this significant attribute could be more effective in the treatment of cancer than drugs that are target specific in their mode of action [72]. In Table 4, we have highlighted a few African plants that have been shown to possess anticancer activity.

### 6.5. Antiviral Activity

Apart from bacterial and fungal infections, the world, and Africa in particular, has to deal with the ruthless devastation of viral diseases, which in most cases are the most difficult to deal with [178]. Unlike bacterial and fungal cells, which exist as free-living entities, viruses are small infectious intracellular parasites, which contain little more than wads of genetic material in the form of either RNA or DNA, surrounded by a lipid-carbohydrate-containing envelope [179]. Viruses can infect all types of organisms, humans, animals, and plants alike. Several antiviral drugs are currently available [180], nonetheless, the high incidence of viral resistance to the existing antiviral drugs has called for an urgent need to search for effective alternative agents [181]. Hence, great attention has been shifted towards screening African plants for novel antiviral agents [182,183]. Antiviral chemotherapy is a standard practice in the management of viral infections in humans [184]. Further research to authenticate the safety of these plants, both in vivo studies and human clinical trials, is necessary. Table 5 lists a few African plants that have been proven to demonstrate antiviral activity.

## 7. Conclusions

Folk medicines are the oldest and most widely utilized out of the different therapeutic systems in African countries and other developing countries. In these countries, the native doctors prescribe easily available herbs to sick people in the rural and semi-urban communities to solve their immediate health problems, and as such, they have utilized several folkloric herbs for different therapeutic purposes. The big question about the safety of the folk medicines will continue to be a major public health issue as their preparation is not hygienic and scientific procedures are not followed. Despite the extensive efforts by researchers, there is still a paucity of information regarding the compilation of plants that can be used in the treatment of several human diseases and their adverse effects. The issues concerning the toxicity of herbal mixtures, appropriate doses to be used, identification of active ingredients, mode(s) of action, quality control measures in herbal preparation and labelling, integration of folkloric and orthodox medicines should be addressed by the relevant authorities to safeguard the life of the populace that explore folk medicines for therapeutic purposes. Awareness about the potential adverse effect, mode of action, and integration of herbal products with orthodox medicines should be encouraged.

## Figures and Tables

**Table 1:** Antibacterial activity of some African flora.

Family	Genus	Part Used	Solvent	Tested Bacterial Strians	Country	Reference
Anacardiaceae	*Mangifera indica* Linn	Stem	Methanol	*Pseudomonas aeruginosa* (isolate); *Escherichia coli* (ATCC 8739); *Staphylococcus aureus* (ATCC 25922); *Proteus mirabilis* (isolate); *Enterococcus faecalis* (ATCC 10541)	Cameroon	[83]
Cactaceae	*Opuntia streptacantha*	Fruit skin	Ethanol and water	*Bacillus subtilis*, *Staphylococcus aureus*, *Micrococcus luteus*, *Salmonella enteritidis*, *Bacillus cereus*, *Klebsiella pneumonia*	Tunisia	[84]
Celastraceae	*Lauridia tetragona*	Leaves	Acetone and methanol	*Enterococcus faecalis* (ATCC 29212), *Staphylococcus aureus* (OK), *Bacillus subtilis* KZN, *Bacillus cereus*, and *Streptococcus pyogenes*, *Vibrio cholera*, *Klebsiella pneumonia* (ATCC 4352), *Pseudomonas aeruginosa* (ATCC 19582), *Salmonella typhi* (OK), and *Escherichia coli* (ATCC 8739)	South Africa	[85]
Fabales	*Elephantorrhiza elephantina*	Rhizome	Methanol	*Bacillus cereus*, *Shigella flexneri*	South Africa	[86]
Zingiberaceae	*Zingiber officinale*	Rhizome	Methanol	*Pseudomonas aeruginosa*, *Salmonella typhi*, *Klebsiella pneumonia*, *Staphylococcus aureus* and *Escherichia coli*	Nigeria	[70]
Vitaceae	*Cissus quadrangularis*	Aerial	Methanol	*Staphylococcus aureus*, *Pseudomonas aeruginosa*, *Escherichia coli*, *Klebsiella pneumonia*	Ethiopia	[87]
Araceae	*Colocasia esculenta*	Whole plant	Methanol	*Pseudomonas aeruginosa, Klebsiella pneumoniae, Enterobacter aerogenes, Escherichia coli* and *Providencia stuartii*	Cameroon	[88]
Asphodelaceae	*Bulbine frutescens*	Whole plant	Methanol	*Escherichia coli*	Kenya	[89]
Salvadoraceae	*Salvadora persica* L.	Bark	Aqueous and methanol	*Staphylococcus aureus* ATCC33862 and *Escherichia coli* ATCC25922	Zimbabwe	[69]
Hypericaceae	*Hypericum roeperianum*	Leaves	Acetone	*Staphylococcus aureus, Enterococcus faecalis and Bacillus cereus, Escherichia coli, Salmonella typhimurium* and *Pseudomonas aeruginosa*	Nigeria	[68]
Phyllanthaceae	*Bridelia ferruginea*	Root	Ethanol	*Escherichia coli* and *Staphylococcus aureus*	Nigeria	[90]
Verbenaceae	*Lippia javanica*	Leaves	Dichloromethane:methanol (1:1)	*Clostridium perfringens*	South Africa	[91]
Agavaceae	*Agave americana*	Leaves	Dichloromethane and methanol (1:1)	*Neisseria gonorrhoeae*	South Africa	[92]
Myrtaceae	*Myrtus nivellei*	Aerial	Butanol	*Bacillus cereus*, *Escherichia coli*, *Listeria monocytogenes*	Algeria	[93]

* ATCC—American Type Culture Collection.

**Table 2:** Antiplasmodial activity of selected African flora.

Family	Species	Part Used	Solvent	Assay Type	Tested Organisms	Country	Reference
Phyllanthaceae	*Phyllanthus amarus*	Whole plant	Aqueous and Ethanol	In vivo	*Plasmodium yoelii*	Nigeria	[99]
Asclepiadaceae	*Periploca linearifolia*	Stem bark	Hexane, chloroform, ethyl acetate and methanol	In vitro	D-6 *Plasmodium falciparum*	Kenya	[100]
Anacardiaceae	*Haematostaphis barteri*	Stem bark	Dichloromethane/methanol	In vivo	*Plasmodium berghei*	Ghana	[101]
Solanaceae	*Withania frutescens*	Leaves and roots	n-hexane	In vitro	*Plasmodium falciparum*	Morocco	[102]
	*Neobegua mahafaliensis*	Leaves	Methanol	In vitro	*Plasmodium falciparum* FcM29-Cameroon	Congo	[103]
Ebenaceae	*Diospyros* species	stem bark	Methanol	In vitro	*Plasmodium falciparum* 3D7	Tanzania	[104]
Euphorbiaceae	*Sebastiania chamaelea*	Whole plant	Methanol	In vitro	*Plasmodium falciparum* FcM29, FcB1, F32 and W2	Niger	[105]
Fabaceae	*Baphia pubescens*	Stem	Hydroethanolic	In vivo	*Plasmodium berghei*	Nigeria	[106]
Burseraceae	*Commiphora africana*	Stem	Dichloromethane and methanol in a 1:1 ratio (*v/v*)	In vitro and in vivo	*Plasmodium falciparum* (D6 and Dd2)	Tanzania	[107]
Zingiberaceae	*Aframomum giganteum*	Stem bark	Dichloromethane and methanol	In vitro	*Plasmodium falciparum* (FCB and W2)	Gabon	[108]
Combretaceae	*Terminalia avicennioides*	leaves	CH_2_Cl_2_, MeOHandMeOH/H_2_O (1/1)	In vitro	*Plasmodium falciparum* strain K1	Burkina Faso	[109]
Meliaceae	*Melia azedarach*	leaves	Cyclohexane, ethyl acetate, dichloromethane, and methanol	In vitro	*Plasmodium falciparum* (3D7 and W2)	Senegal	[110]
Asteraceae	*Bidens pilosa*	leaves	Methanolic and Ethyl Acetate	In vitro	*Plasmodium falciparum* (Pf3D7 and PfINDO)	Cameroon	[111]
Labiatae	*Ajuga remota*	leaves	Hydroethanolic	In vivo	*Plasmodium berghei* (ANKA strain)	Ethiopia	[112]
Euphorbiaceae	*Alchornea laxiflora*	leaves	Ethanol	In vivo	*Plasmodium falciparum* (Pf 3D7) and (Pf INDO)	Nigeria	[113]
Compositae	*Vernonia adoensis*	Leaves	Aqueous, methanol and chloroform	In vivo	*Plasmodium berghei*	Ethiopia	[114]
Lamiaceae	*Clerodendrum rotundifolium*	Leaves	ethyl acetate	In vitro	*Plasmodium falciparum* (NF54 and FCR3)	Uganda	[115]
Apocynaceae	*Tabernaemontana elegans*	Stem bark	Dichloromethane: 50% methanol (1:1)	In vitro	*Plasmodium falciparum* (NF54)	South Africa	[96]
Zygophyllaceae	*Balanites aegyptiaca*	aerial	Methanol	In vitro	*Plasmodium falciparum*	Togo	[116]
Caesalpiniaceae	*Cassia occidentalis*	Leaves	Ethanol	In vitro	*Plasmodium falciparum*	Congo	[117]
Fabaceae	*Pericopsis laxiflora*	Bark	Ethanol	In vitro	*Plasmodium falciparum* (NF54) and K1)	Côte d’Ivoire	[118]
Canellaceae	*Warburgia salutaris*	stem bark	Dichloromethane	In vitro and in vivo	NF54 *Plasmodium falciparum*	South Africa	[119]
Asterales	*Oedera genistifolia*	leaves	Chloroform, ethyl acetate, and ethanol	In vitro	*Plasmodium falciparum* strain 3D	South Africa	[120]

**Table 3:** Antifungal activity of some African flora.

Family	Genus	Part Used	Solvent	Tested Organisms	Country	Reference
Asteraceae	*Scolymus hispanicus*	Leaves and stem	Ethanol and dichloromethane	*Candida neoformans* and *Candida albicans*	Morocco	[127]
Fabaceae	*Prosopis juliflora*	Whole plant	Methanol or water	*Colletotrichum musae*	Ethiopia	[128]
Asphodelaceae	*Aloe barbadensis*	leaves	Ethanol	*Candida albicans* and *Tessaracoccus flavus*	Ghana	[129]
Lythraceae	*Punica granatum*	Fruits	Ethanol	*Fusarium oxysporum, Fusarium culmorum, Fusarium graminearum, Aspergillus niger* and *Alternaria alternata*	Tunisia	[130]
Celastraceae	*Mystroxylon aethiopicum*	Leave, stems and roots	Dichloromethane and ethyl acetate	*Candida albicans* and *Candida neoformans*	Tanzania	[131]
Canellaceae	*Warburgia ugandensis*	Leaves	Water, acetone, ethanol, and hexane	*Candida albicans* SC5314, *Candida glabrata* BG2, *Candida glabrata* ATCC 2001	Kenya	[132]
Lamiaceae	*Gmelina arborea*	seeds	Water	*Athelia rolfsii* and *Sclerotium rolfsii*	Nigeria	[133]
Ranunculaceae	*Clematis flammula*	leaves and bark	Ethanol	*Candida albicans*	Algeria	[134]
Lamiaceae	*Marrubium vulgare*	aerial	Methanol and acetone	*Botrytis cinerea, Pythium ultimum*	Tunisia	[135]
Asteraceae	*Artemisia herba alba*	aerial	Water	*Usarium graminearum* (ITEM-6477) and *Fusarium sporotrichioides* (ITEM-692)	Algeria	[136]
Euphorbiaceae	*Ricinus communis*	Leaves	Aqueous, methanol and ethanol	*Candida albicans*	Ghana	[137]
Lecythidaceae	*Barringtonia asiatica*	Leaves	Methanol	*Aspergillus niger, Aspergillus flavin, Candida tropicalis* and *Fusarium oxysporium*	Nigeria	[138]
Asparagaceae	*Agave sisalana*	Roots	Acetone, n-hexane, dichloromethane and methanol	*Candida albicans*, *Candida glabrata*, *Candida krusei*, *Candida parapsilosis*, *Candida tropicalis* and *Cryptococcus neoformans*	South Africa	[139]

**Table 4:** Anticancer activity of some African flora.

Family	Genus	Part Used	Solvent	Tested Organisms	Country	Reference
Curtisiaceae	*Curtisia dentata*	Leaves	Acetone	MCF-7, colorectal carcinoma cells (Caco-2), A549 and HeLa	South Africa	[73]
Lamiaceae	*Origanum compactum*	leaves	n-hexane	L20B, RD and Vero	Morocco	[162]
Liliaceae	*Allium ascalonicum*	Tuber	Methanol	MCF-7, MDA-MB-231 and fibroblast cells	Nigeria	[163]
Asparagales	*Boophone disticha*	Bulb	Chloroform, acetone, and ethanol	HeLa	South Africa	[164]
Annonaceae	*Uvariodendron anisatum*	Root	Methanol and water	4T1 breast cancer cell line	Kenya	[165]
Lauraceae	*Beilschmiedia acuta Kosterm,*	roots, leaves, bark	Methanol	Leukemia CCRF-CEM	Cameroon	[72]
Aristolochiaceae	*Aristolochia ringens* Vahl.	Root	Ethanol and water	A549 (lung), HCT-116 (colon), PC3 (prostate), A431 (skin), HeLa (cervix), and THP-1 (leukemia)	Nigeria	[166]
Fabaceae	*Acacia nilotica* (L.) Delile	Fruit	Dichloromethane or hexane with chloroform or ethyl acetate or ethanol or methanol 80%	Human leukemic CCRF-CEM and the ABCB1 (P-gp)	Sudan	[167]
Euphorbiaceae	*Jatropha zeyheri*	roots	Ethyl acetate	Human dermal fibroblast (HDF), colon adenocarcinoma (Caco-2), lung cancer (A547) and breast cancer (MCF-7)	South Africa	[168]
Poaceae	*Cymbopogon schoenanthus* L. Spreng	Aerial	Ethanol	MCF-7	Tunisia	[169]
Malvaceae	*Hibiscus sabdariffa*	Whole plant	Methanol	PC3 (prostate cancer cell line)	Sudan	[170]
Anacardiaceae	*Rhus tripartita*	Aerial	Methanol 70% and acetone 70%	CaCo-2 (colon carcinoma) and K-562 (myelogenous leukemia)	Tunisia	[171]
Myrtales	*Syzygium jambos*	Leaves	Ethanol	HeLa, Vero and HEK-293	South Africa	[172]
Bignoniaceae	*Jacaranda copaia*	leaves, bark, flower, root,	Ethanol	MDA-MB231 (breast) and PANC-1 (pancreas)	Venezuela	[173]
Asteraceae	*Ageratum conyzoides*	whole plant and parts - stem, leaves and flower	50% hydroethanolic	Jurkat, LNCap, MCF7	Ghana	[174]
Clusiaceae	*Pentadesma butyracea*	Fruits	Methanol	Skin cancer: A431	Cameroon	[175]
Sapotaceae	*Sideroxylon oxyacanthum*	Leaves	80% methanol (MeOH) in H_2_O	MT-1, MCF-7 and MCF-10A	Ethiopia	[176]
Chrysobalanaceae	*Parinari curatellifolia* (Planch ex Benth)	leaves, roots stem and bark	Methanol	Wil 2 and Jurkat T	Zimbabwe	[177]

**Table 5:** Antiviral activity of some African flora.

Family	Genus	Part Used	Solvent	Tested Organisms	Country	Reference
Myrtaceae	*Syzygium jambos*	Leaves	Ethanol	HSV-1	South Africa	[172]
Euphorbiaceae	*Macaranga barteri*	Leaves	Methanol	Echoviruses (E7 and and E19)	Nigeria	[185]
Meliaceae	*Trichilia dregeana*	Roots	Methanol	hepatitis C virus (HCV)	Cameroon	[186]
Celastraceae	*Cassine transvaalensis*	Bark	Methanol	HIV	South Africa	[187]
Fabaceae	*Caesalpinia decapetala*	Root	Water	HSV-1	Kenya	[188]
Apocynaceae	*Carissa edulis*	Root	Water	HSV-1 and HSV-2	Kenya	[189]
Fabaceae	*Cajanus cajan*	Leaves, stem, roots	Hot water	HSV-1	Nigeria	[190]
Poaceae	*Bambusa vulgaris*	Leaf	Ethanol	Measles and yellow fever virus	Nigeria	[191]
Combretaceae	*Combretum molle*	Roots	Methanol	HIV-1	South Africa	[192]
Amaryllidaceae	*Zephyranthes candida*	Stem bark and root bark	methanol	Poliovirus Type-PV1	Nigeria	[185]
Apocynaceae	*Tabernaemontana ventricosa*	Leaves	Methanol	influenza A virus (IAV)	South Africa	[183]
Amaryllidaceae	*Crinum macowani*	Leaves	Aqueous and ethanol	HIV	South Africa	[193]
Malvaceae	*Hibiscus sabdariffa*	Leaves	Ethanol	Measles Virus	Nigeria	[194]

## Data Availability

Not applicable.

## References

[1] Mahomoodally F.M. (2013). Traditional medicines in Africa: An appraisal of ten potent African medicinal plants. Evid. Based Complement. Altern. Med..

[2] Manach C., Scalbert A., Morand C., R’em´esy C., Jim´enez L. (2004). Polyphenols: Food sources and bioavailability. Am. J. Clin. Nutr..

[3] World Health Organisation (2004). Guidelines on Safety Monitoring of Herbal Medicines in Pharmacovigilance Systems. http//apps.who.int/medicinedocs/documents/s14215e.

[4] World Health Organisation (2013). A Global Brief on Hypertension: World Health Day. http://www.who.int.

[5] World Health Organization (2008). Traditional Medicines. http://www.who.int/mediacentre/factsheets/fs134/en/.

[6] Bandaranayake W.M. (2006). Quality Control, Screening, Toxicity, and Regulation of Herbal Drugs. Modern Phytomedicine: Turning Medicinal Plants into Drugs.

[7] Bodeker C., Bodeker G., Ong C.K., Grundy C.K., Burford G., Shein K. (2005). WHO Global Atlas of Traditional, Complementary and Alternative Medicine.

[8] Frenzel C., Teschke R. (2016). Herbal hepatotoxicity: Clinical characteristics and listing compilation. Int. J. Mol. Sci..

[9] Fokunang C.N., Ndikum V., Tabi O.Y., Jiofack R.B., Ngameni B., Guedje N.M., Tembe-Fokunang E.A., Tomkins P., Barkwan S., Kechia F. (2011). Traditional medicine: Past, present and future research and development prospects and integration in the National Health System of Cameroon. Afr. J. Tradit. Complement. Altern. Med..

[10] Gurib-Fakim A. (2006). Medicinal plants: Traditions of yesterday and drugs of tomorrow. Mol. Asp. Med..

[11] Fennell C.W., Light M.E., Sparg S.G., Stafford G.I., van Staden J. (2004). Assessing African medicinal plants for efficacy and safety: Agricultural and storage practices. J. Ethnopharmacol..

[12] Mphasha L.E., Lebese R.T. (2015). The importance of indigenous languages in health-care services: Some observations from Limpopo Province, South Africa. Stud. Ethno Med..

[13] Ekor M. (2014). The growing use of herbal medicines: Issues relating to adverse reactions and challenges in monitoring safety. Front. Pharmacol..

[14] Yuan H., Ma Q., Ye L., Piao G. (2016). The traditional medicine and modern medicine from natural products. Molecules.

[15] Mothibe M.E., Sibanda M. (2019). African traditional medicine: South African perspective. Traditional and Complementary Medicine.

[16] Ozioma E.O.J., Chinwe O.A.N. (2019). Herbal medicines in African traditional medicine. Herb. Med..

[17] Agyemang-Duah W., Peprah C., Peprah P. (2019). Barriers to formal healthcare utilisation among poor older people under the livelihood empowerment against poverty programme in the Atwima Nwabiagya District of Ghana. BMC Public Health.

[18] Parle M., Bansal N. (2006). Herbal medicines: Are they safe?. Nat. Prod. Radiance.

[19] World Health Organization (2003). Traditional Medicine, Highlights of the 56th World Health Assembly. www.who.int/features/2003/05b/en.

[20] World Health Organisation (2011). Regional Office for Africa, Progress Report on Decade of Traditional Medicine in the African Region. www.afro.who.int/.

[21] World Health Organisation (2012). The WHO Traditional Medicine Strategy 2014–2023. https://www.who.int/medicines/publications/traditional/trm_strategy14_23/en/.

[22] Calapai G. (2008). European legislation on herbal medicines: A look into the future. Drug Saf..

[23] Anquez-Traxler C. (2011). The legal and regulatory framework of herbal medicinal products in the European Union: A focus on the traditional herbal medicines category. Drug Inf. J..

[24] Kong J.M., Goh N.K., Chia L.S., Chia T.F. (2003). Recent advances in traditional plant drugs and orchids. Acta Pharmacol. Sin..

[25] Govender S., Du Plessis-Stoman D., Downing T.G., van de Venter M. (2006). Traditional herbal medicines: Microbial contamination, consumer safety and the need for standards. S. Afr. J. Sci..

[26] Kosalec I., Cvek J., Tomić S. (2009). Contaminants of medicinal herbs and herbal products. Arch. Ind. Hyg. Toxicol..

[27] van Vuuren S., Williams V.L., Sooka A., Burger A., van der Haar L. (2014). Microbial contamination of traditional medicinal plants sold at the faraday muthi market, Johannesburg, South Africa. S. Afr. J. Bot..

[28] Rietjens I.M., Martena M.J., Boersma M.G., Spiegelenberg W., Alink G.M. (2005). Molecular mechanisms of toxicity of important food-borne phytotoxins. Mol. Nutr. Food Res..

[29] Teschke R., Wolff A., Frenzel C., Schulze J., Eickhoff A. (2012). Herbal hepatotoxicity A tabular compilation of reported cases. Liver Int..

[30] Seeff L.B., Bonkovsky H.L., Navarro V.J., Wang G. (2015). Herbal products and the liver: A review of adverse effects and mechanisms. Gastroenterology.

[31] Omage K., Azeke M.A., Orhue J.N., Iseghohi S.O. (2017). Toxicological implications of the therapeutic use of Acalypha wilkesiana leaves in traditional medicine. Clin. Phytosci..

[32] Iroezindu M.O., Agbaji O.O., Daniyam C.A., Isiguzo G.C., Isichei C., Akanbi M.O. (2013). Liver function test abnormalities in Nigerian patients with human immunodeficiency virus and hepatitis B virus co-infection. Int. J. STD AIDS.

[33] Amadi C.N., Orisakwe O.E. (2018). Herb-induced liver injuries in developing nations: An update. Toxics.

[34] Ogutcu A., Suludere Z., Kalendar Y. (2008). Dichlorvos-induced hepatotoxicity in rats and the protective effects of vitamins C and E. Environ. Toxicol. Pharmacol..

[35] Chang S.Y., Voellinger J.L., van Ness K.P., Chapron B., Shaffer R.M., Neumann T., White C.C., Kavanagh T.J., Kelly E.J., Eaton D.L. (2017). Characterization of rat or human hepatocytes cultured in microphysiological systems (MPS) to identify hepatotoxicity. Toxicol. In Vitro.

[36] Pfeiffer J.M., Butz R.J. (2005). Assessing cultural and ecological variation in ethnobiological research: The importance of gender. J. Ethnobiol..

[37] Lamxay V., de Boer H.J., Björk L. (2011). Traditions and plant use during pregnancy, childbirth and postpartum recovery by the Kry ethnic group in Lao PDR. J. Ethnobiol. Ethnomed..

[38] Titilayo O.K., Rasaq A., Ismail E.M. (2009). Attitude and use of herbal medicines among pregnant women in Nigeria. BMC Complement. Altern. Med..

[39] Zumsteg I.S., Weckerle C.S. (2007). Bakera, a herbal steam bath for postnatal care in Minahasa (Indonesia): Documentation of the plants used and assessment of the method. J. Ethnopharmacol..

[40] Adane F., Seyoum G., Alamneh Y.M., Abie W., Desta M., Sisay B. (2020). Herbal medicine use and predictors among pregnant women attending antenatal care in Ethiopia: A systematic review and meta-analysis. BMC Pregnancy Childbirth.

[41] Veale D.J.H., Furman K.I., Oliver D.W. (1992). South African traditional herbal medicines used during pregnancy and childbirth. J. Ethnopharmacol..

[42] Sarkar M., Chaudhuri G.R., Chattopadhayay A., Biswas N.M. (2003). Effect of sodium arsenita on spermatogenesis, plasma gonadotrophins and testosterone in rats. Asian J. Androl..

[43] Yakubu T.M., Ajiboye O.T., Adewunmi A.M. (2014). Toxicity and beneficial effects of some african plants on the reproductive system. Toxicological Survey of African Medicinal Plants.

[44] Ait H.N., Slimani M., Aous A.E.K. (2009). Reproductive toxicity of lead acetate in albinos’ rat. Am. J. Sci. Res..

[45] Cinar O., Simez O., Can A. (2015). Carbofuran alters centrosome and spindle organization, and delays cell division in oocytes and mitotic cells. Toxicol. Sci..

[46] Sorelle D.N., Ferdinand N., Vemo Bertin Narcisse T. (2019). Medicinal plants and female reproduction disorders due to oxidative stress. Arch. Vet. Sci..

[47] Heikal T.M., Mossa T.H., Marei G.I.K.H., Abdel Rasoul M.A. (2012). Cyromazine and chlorpyrifos induced renal toxicity in rats: The ameliorating effects of green tea extract. J. Anal. Toxicol..

[48] Doltade S., Lonare M., Raut S., Telang A. (2019). Evaluation of acetamiprid mediated oxidative stress and pathological changes in male rats: Ameliorative effect of curcumin. Proc. Natl. Acad. Sci. USA India Sect. B Biol. Sci..

[49] Sangha G.K., Kaur K., Khera K.S. (2013). Cypermethrin induced pathological and biochemical changes in reproductive organs of female rats. J. Environ. Biol..

[50] Nwangwa E.K. (2012). Antifertility effects of ethanolic extract of *Xylopia aethiopica* on male reproductive organ of wistar rats. Am. J. Med. Sci..

[51] Akbarsha M.A., Mavivannan B., Hamid K.S., Vijayan B. (1990). Antifertility effect of *Andrographis paniculata* (Nees) in albino rats. Indian J. Exp. Biol..

[52] Nergard C.S., Ho T.P.T., Diallo D., Ballo N., Paulsen B.S., Nordeng H. (2015). Attitudes and use of medicinal plants during pregnancy among women at health care centers in three regions of Mali, West-Africa. J. Ethnobiol. Ethnomed..

[53] Nyeko R., Tumwesigye N.M., Halage A.A. (2016). Prevalence and factors associated with use of herbal medicines during pregnancy among women attending postnatal clinics in Gulu district, Northern Uganda. BMC Pregnancy Childbirth.

[54] Hill J., Kayentao K., Achieng F., Diarra S., Dellicoour S., Diawara S.I., Hamel M.J., Ouma P., Desai M., Doumbo O.K. (2015). Access and use of interventions to prevent and treat malaria among pregnant women in Kenya and Mali: A qualitative study. PLoS One.

[55] Shewamene Z., Dune T., Smith C.A. (2017). The use of traditional medicine in maternity care among African women in Africa and the diaspora: A systematic review. BMC Complement. Altern. Med..

[56] Moreira D.D.L., Teixeira S.S., Monteiro M.H.D., de-Oliveira A.C.A., Paumgartten F.J. (2014). Traditional use and safety of herbal medicines. Rev. Bras. Farmacogn..

[57] Zhou J., Ouedraogo M., Qu F., Duez P. (2013). Potential genotoxicity of traditional Chinese medicinal plants and phytochemicals: An overview. Phytother. Res..

[58] de Sousa Lima C.M., Fujishima M.A.T., de Paula Lima B., Mastroianni P.C., de Sousa F.F.O., da Silva J.O. (2020). Microbial contamination in herbal medicines: A serious health hazard to elderly consumers. BMC Complement. Altern. Med..

[59] Tugume P., Nyakoojo C. (2019). Ethno-pharmacological survey of herbal remedies used in the treatment of paediatric diseases in Buhunga parish, Rukungiri District, Uganda. BMC Complement. Altern. Med..

[60] Sasidharan S., Saravanan D., Chen Y., Sundram K.M., Latha L.Y. (2011). Extraction, isolation and characterization of bioactive compounds from plants’ extracts. Afr. J. Tradit. Complement. Altern. Med..

[61] Mensah M.L., Komlaga G., Forkuo A.D., Firempong C., Anning A.K., Dickson R.A. (2019). Toxicity and safety implications of herbal medicines used in Africa. Herb. Med..

[62] Muhammad B.Y., Awaisu A. (2008). The need for enhancement of research, development, and commercialization of natural medicinal products in Nigeria: Lessons from the Malaysian experience. Afr. J. Tradit. Complement. Altern. Med..

[63] Manilal A., Sabu K.R., Shewangiza M., Aklilu A., Seid M., Merdekios B., Tsegaye B. (2020). In vitro antibacterial activity of medicinal plants against biofilm-forming methicillin-resistant *Staphylococcus aureus*: Efficacy of *Moringa stenopetala* and *Rosmarinus officinalis* extracts. Heliyon.

[64] Berdy J. (2005). Bioactive microbial metabolites. J. Antibiot..

[65] Manilal A., Sujith S., Sabarathnam B., Selvin J., Shakir C., Lipton A.P. (2011). Biological activity of the red alga, *Laurencia brandenii*. Acta Bot. Croat..

[66] Samuelsson G. (2004). Drugs of Natural Origin: A Textbook of Pharmacognosy.

[67] Stefanovic O.D. (2018). Synergistic Activity of Antibiotics and Bioactive Plant Extracts: A Study Against Gram-Positive and Gram-Negative Bacteria. Bacterial Pathogenesis and Antibacterial Control.

[68] Elisha L.L., Botha F.S., McGaw L.J., Eloff J.N. (2017). The antibacterial activity of extracts of nine plant species with good activity against *Escherichia coli* against five other bacteria and cytotoxicity of extracts. BMC Complement. Altern. Med..

[69] Mudzengia C.P., Murwiraa A., Tivapasic M.T., Murungwenid C., Joan V., Burumue V.B., Halimanif T. (2017). Antibacterial activity of aqueous and methanol extracts of selected species used in livestock health management. Pharm. Biol..

[70] Yusuf A.A., Lawal B., Abubakar A.N., Berinyuy E.B., Omonije Y.O., Umar S.I., Shebe M.N., Alhaji Y.M. (2018). In-vitro antioxidants, antimicrobial and toxicological evaluation of Nigerian Zingiber officinale. Clin. Phytosci..

[71] Leonti M., Dal Cero M., Weckerle C.S., Laura C. Reverse Etnopharmacology and drug discovery. Proceedings of the 15th International Congress of the International Society for Ethnopharmacology.

[72] Kuete V., Tankeo S.B., Saeed M.E., Wienc B., Tane P., Efferth T. (2014). Cytotoxicity and modes of action of five Cameroonian medicinal plants against multi-factorial drug resistance of tumor cells. J. Ethnopharmacol..

[73] Soyingbe O.S., Mongalo N.I., Makhafola T.J. (2018). In vitro antibacterial and cytotoxic activity of leaf extracts of *Centella asiatica* (L.) Urb, *Warburgia salutaris* (Bertol. F.) Chiov and *Curtisia dentata* (Burm. F.) CA Sm-medicinal plants used in South Africa. BMC Complement. Altern. Med..

[74] Idris O.A., Wintola O.A., Afolayan A.J. (2019). Evaluation of the bioactivities of *Rumex crispus* L. leaves and root extracts using toxicity, antimicrobial, and antiparasitic assays. Evid. Based Complement. Altern. Med..

[75] Kuete V., Kuete V. (2013). Medicinal plant research in Africa. Pharmacology and Chemistry.

[76] Okach D., Nyunja A., Opande G. (2013). Phytochemical screening of some wild plants from Lamiaceae and their role in traditional medicine in Uriri district-Kenya. Int. J. Herb. Med..

[77] Ortíz-Castro R., Contreras-Cornejo H.A., Macías-Rodríguez L., López-Bucio J. (2009). The role of microbial signals in plant growth and development. Plant. Signal. Behav..

[78] Ayaz M., Ullah F., Sadiq A., Ulla F., Ovais M., Ahmed J., Devkota H.P. (2019). Synergistic interactions of phytochemicals with antimicrobial agents: Potential strategy to counteract drug resistance. Chem. Biol. Interact..

[79] Nguta J.M., Kiraithe M.N. (2019). In vitro antimicrobial activity of aqueous extracts of *Ocimum suave* Willd., *Plectranthus barbatus* andrews and *Zanthoxylum chalybeum* Engl. against selected pathogenic bacteria. Biomed. Biotechnol. Res. J..

[80] Ali S.S., Ayub A., Ali S.N., Begum S., Siddiqui B.S., Mahmood M., Khan K.L. (2017). Antibacterial activity of methanolic extracts from some selected medicinal plants FUUAST. J. Biol..

[81] Deshpande B., Varsha C., Bhawana P. (2017). Antibacterial activity of plant extract of *Amaranthus spinosus*. Indian J. Scientometr. Res..

[82] Ogbunugafor H.A., Ugochukwu C.G., Kyrian-Ogbonna A.E. (2017). The role of spices in nutrition and health: A review of three popular spices used in southern Nigeria. Food Qual. Saf..

[83] Tchoumi L.T., Nchouwet M.L., Kaman S.P., Nana W.Y., Djimeli R.D., Kamanyi A., Ngnokam S.W. (2020). Antimicrobial and antidiarrhoeal activities of aqueous and methanolic extracts of *Mangifera indica* Linn stem bark (Anarcadiaceae) in Wistar rats. Adv. Tradit. Med..

[84] Zourgui M.N., Hfaiedh M., Brahmi D., Affi W., Gharsallah N., Zourgui L., Amri M. (2020). Phytochemical screening, antioxidant and antimicrobial activities of *Opuntia streptacantha* fruit skin. J. Food Meas. Charact..

[85] Wintola O.A., Afolayan A.J. (2019). Phytochemical and antimicrobial evaluation of *Lauridia tetragona* (LF) RH Archer A medicinal plant used for the management of dysentery in the Eastern Cape Province of South Africa. Pharmacogn. Res..

[86] Asong J.A., Amoo S.O., McGaw L.J., Nkadimeng S.M., Arem A.O., Otang-Mbeng W. (2019). Antimicrobial activity, antioxidant potential, cytotoxicity and phytochemical profiling of four plants locally used against skin diseases. Plants.

[87] Mummed B., Abraha A., Feyera T., Nigusse A., Assefa S. (2018). In vitro antibacterial activity of selected medicinal plants in the traditional treatment of skin and wound infections in Eastern Ethiopia. BioMed Res. Int..

[88] Dzotam J.K., Touani F.K., Kuete V. (2016). Antibacterial activities of the methanol extracts of *Canarium schweinfurthii* and four other Cameroonian dietary plants against multi-drug resistant Gram-negative bacteria. Saudi J. Biol. Sci..

[89] Opinde H.R., Gatheri G.W., Nyamache A.K. (2016). Antimicrobial evaluation of crude methanolic leaf extracts from selected medicinal plants against *Escherichia coli*. J. Bacteriol. Parasitol..

[90] Yunana B.T., Guiyi J.C., Bukar B.B. (2018). In vitro and in vivo evaluation of antibacterial activity of *Bridelia ferrugine* extracts on some clinical isolates. J. Phytopharmacol..

[91] Shirinda H., Leonard C., Candy G., van Vuuren S. (2019). Antimicrobial activity and toxicity profile of selected southern African medicinal plants against neglected gut pathogens. S. Afr. J. Sci..

[92] Seleteng-Kose L., Moteetee A., van Vuuren S. (2019). Medicinal plants used for the treatment of sexually transmitted infections in the Maseru District, Lesotho: Antimicrobial validation, phytochemical and cytotoxicity studies. S. Afr. J. Bot..

[93] Ramdane F., Essid R., Fares N., El Ouassis D., Aziz S., Mahammed M.H., Hadj M.D.O., Limam F. (2017). Antioxidant antileishmanial cytotoxic and antimicrobial activities of a local plant *Myrtus nivellei* from Algeria Sahara. Asian Pac. J. Trop. Biomed..

[94] Kigondu E.V.M., Rukunga G.M., Gathirwa J.W., Irungu B.N., Mwikwabe N.M., Amalemba G.M., Omar S.A., Kirira P.G. (2011). Antiplasmodial and cytotoxicity activities of some selected plants used by the Maasai community, Kenya. S. Afr. J. Bot..

[95] Bekono B.D., Ntie-Kang F., Onguéné P.A., Lifong L.L., Sippl W., Fester K., Owono L.C. (2020). The potential of anti-malarial compounds derived from African medicinal plants: A review of pharmacological evaluations from 2013 to 2019. Malar. J..

[96] Bapela M.J., Meyer J.M., Kaiser M. (2014). In vitro antiplasmodial screening of ethnopharmacologically selected South African plant species used for the treatment of malaria. J. Ethnopharmacol..

[97] Ahmed E.H.M., Nour B.Y., Mohammed Y.G., Khalid H.S. (2010). Antiplasmodial activity of some medicinal plants used in Sudanese folk-medicine. Environ. Health Insights.

[98] Onguéné P.A., Ntie-Kang F., Lifongo L.L., Ndom J.C., Sippl W., Mbaze L.M.A. (2013). The potential of anti-malarial compounds derived from African medicinal plants. Part I: A pharmacological evaluation of alkaloids and terpenoids. Malar. J..

[99] Ajala T.O., Igwilo C.I., Oreagba I.A., Odeku O.A. (2011). The antiplasmodial effect of the extracts and formulated capsules of *Phyllanthus amarus* on *Plasmodium yoelii* infection in mice. Asian Pac. J. Trop. Med..

[100] Ayuko T.A., Njau R.N., Cornelius W., Leah N., Ndiege I.O. (2009). In vitro antiplasmodial activity and toxicity assessment of plant extracts used in traditional malaria therapy in the Lake Victoria Region. Memórias Inst. Oswaldo Cruz.

[101] Boampong J.N., Karikari A.A., Ameyaw E.O. (2015). In vivo antiplasmodial and in vitro antioxidant properties of stem bark extracts of *Haematostaphis barteri*. Asian Pac. J. Trop. Biomed..

[102] El Bouzidi L., Bakrim W.B., Mahiou V., Azas N., Larhsini M., Markouk M., Ollivier E., Bekkouche K. (2017). In vitro antiplasmodial activity of *Withania frutescens*—Solanaceae. Eur. J. Integr. Med..

[103] Ngbolua K.N., Rafatro H., Rakotoarimanana H., Ratsimamanga U.S., Mudogo V., Mpiana P.T., Tshibangu D.S.T. (2011). Pharmacological screening of some traditionally used antimalarial plants from the Democratic Republic of Congo compared to their ecological taxonomic equivalence in Madagascar. Int. J. Biol. Chem..

[104] Christopher R., Mgani Q.A., Nyandoro S.S., Rousseau A.L., van Vuuren S.F., Isaacs M., Hoppe H.C. (2018). Antitrypanosomal, antiplasmodial, and antibacterial activities of extracts from selected *Diospyros* and *Annonaceae* species. J. Complement. Med. Res..

[105] Garcia-Alvarez M.C., Moussa I., Soh P.N., Nongonierma R., Abdoulaye A., Nicolau-Travers M.L., Fabre A., Wdzieczak-Bakala J., Ahond A., Poupat C. (2013). Both plants *Sebastiania chamaelea* from Niger and *Chrozophora senegalensis* from Senegal used in African traditional medicine in malaria treatment share a same active principle. J. Ethnopharmacol..

[106] Ihekwereme C.P., Agbata C.A., Chukwueze K.O., Agu S.C. (2016). In vivo evaluation of antiplasmodial activity of hydroethanolic stem extract of *Baphia pubescens* in *Plasmodium berghei* infected albino mice. J. HerbMed Pharmacol..

[107] Kweyamba P.A., Zofou D., Efange N., Assob J.C.N., Kitau J., Nyindo M. (2019). In vitro and in vivo studies on anti-malarial activity of *Commiphora africana* and *Dichrostachys cinerea* used by the Maasai in Arusha region, Tanzania. Malar. J..

[108] Lekana-Douki J.B., Bongui J.B., Liabagui S.L.O., Edou S.E.Z., Zatra R., Bisvigou U., Druilhe P., Lebibi J., Ndouo F.S.T., Kombila M. (2011). In vitro antiplasmodial activity and cytotoxicity of nine plants traditionally used in Gabon. J. Ethnopharmacol..

[109] Ouattara L.P., Sanon S., Mahiou-Leddet V., Gansané A., Baghdikian B., Traoré A., Nébié I., Traoré A.S., Azas N., Ollivier E. (2014). In vitro antiplasmodial activity of some medicinal plants of Burkina Faso. Parasitol. Res..

[110] Manga A., Gassama A., Diatta K., Bassène E., Cojean S., Cavé C. (2018). Antiplasmodial activity of extracts OF *Khaya senegalensis* (DERS.) A. Jus (Meliaceae) and *Melia azedarach* L.; Plants of Senegalese Traditional Medicine. Int. J. Pharm. Sci. Res..

[111] Zofou D., Tene M., Ngemenya M.N., Tane P., Titanji V.P. (2011). In vitro antiplasmodial activity and cytotoxicity of extracts of selected medicinal plants used by traditional healers of Western Cameroon. Malar. Res. Treat..

[112] Nardos A., Makonnen E. (2017). In vivo antiplasmodial activity and toxicological assessment of hydroethanolic crude extract of *Ajuga remota*. Malar. J..

[113] Okokon J.E., Obot A.U., Mohanakrishnan D., Mittal G., Sahal D. (2017). Antimalarial and antiplasmodial activity of leaf extract of *Alchornea laxiflora*. J. Herbs Spices Med. Plants.

[114] Zemicheal G., Mekonnen Y. (2018). Antiplasmodial activity of *Vernonia adoensis* aqueous, methanol and chloroform leaf extracts against chloroquine sensitive strain of Plasmodium berghei in vivo in mice. BMC Res. Notes.

[115] Adia M.M., Emami S.N., Byamukama R., Faye I., Borg-Karlson A.K. (2016). Antiplasmodial activity and phytochemical analysis of extracts from selected Ugandan medicinal plants. J. Ethnopharmacol..

[116] Karou S.D., Tchacondo T., Ouattara L., Anani K., Savadogo A., Agbonon A., Attaia M.B., de Souza C., Sakly M., Simpore J. (2011). Antimicrobial, antiplasmodial, haemolytic and antioxidant activities of crude extracts from three selected Togolese medicinal plants. Asian Pac. J. Trop. Dis..

[117] Tona L., Cimanga R.K., Mesia K., Musuamba C.T., de Bruyne T., Apers S., Hernans N., van Miert S., Pieters L., Totté J. (2004). In vitro antiplasmodial activity of extracts and fractions from seven medicinal plants used in the Democratic Republic of Congo. J. Ethnopharmacol..

[118] Koffi J.A., Silué K.D., Tano D.K., Dable T.M., Yavo W. (2020). Evaluation of antiplasmodial activity of extracts from endemic medicinal plants used to treat malaria in Côte d’Ivoire. BioImpacts BI.

[119] Nyaba Z.N., Murambiwa P., Opoku A.R., Mukaratirwa S., Shode F.O., Simelane M.B. (2018). Isolation, characterization, and biological evaluation of a potent anti-malarial drimane sesquiterpene from *Warburgia salutaris* stem bark. Malar. J..

[120] Okaiyeto K., Okoh A.I. (2020). In vitro assessment of antiplasmodial and antitrypanosomal activities of chloroform, ethyl acetate and ethanol leaf extracts of *Oedera genistifolia*. Appl. Sci..

[121] Sales M.D.C., Costa H.B., Fernandes P.M.B., Ventura J.A., Meira D.D. (2016). Antifungal activity of plant extracts with potential to control plant pathogens in pineapple. Asian Pac. J. Trop. Biomed..

[122] Samie A., Mashau F. (2013). Antifungal activities of fifteen Southern African medicinal plants against five *Fusarium* species. J. Med. Plants Res..

[123] Lall N., Kishore N. (2014). Are plants used for skin care in South Africa fully explored?. J. Ethnopharmacol..

[124] Lewu F.B., Afolayan A.J. (2009). Ethnomedicine in South Africa: The role of weedy species. Afr. J. Biotechnol..

[125] Arif T., Bhosale J.D., Kumar N., Mandal T.K., Bendre R.S., Lavekar G.S., Dabur R. (2009). Natural products-antifungal agents derived from plants. J. Asian Nat. Prod. Res..

[126] Mahlo S.M., McGaw L.J., Eloff J.N. (2010). Antifungal activity of leaf extracts from South African trees against plant pathogens. Crop. Prot..

[127] Aboukhalaf A., El Amraoui B., Tabatou M., da Rocha J.M.F., Belahsen R. (2020). Screening of the antimicrobial activity of some extracts of edible wild plants in Morocco. Funct. Food Health Dis..

[128] Bazie S., Ayalew A., Woldetsadik K. (2014). Antifungal activity of some plant extracts against (*Colletotrichum musae*) the cause of postharvest banana anthracnose. J. Plant Pathol. Microbiol..

[129] Donkor A.M., Donkor M.N., Kuubabongnaa N. (2020). Evaluation of anti-infective potencies of formulated aloin A ointment and aloin A isolated from *Aloe barbadensis* Miller. BMC Chem..

[130] Hlima H.B., Bohli T., Kraiem M., Ouederni A., Mellouli L., Michaud P., Abdelkafi S., Smaoui S. (2019). Combined effect of *Spirulina platensis* and *Punica granatum* peel extracts: Phytochemical content and antiphytophatogenic activity. Appl. Sci..

[131] Kilonzo M., Ndakidemi P.A., Chacha M. (2016). In vitro antifungal and cytotoxicity activities of selected Tanzanian medicinal plants. Trop. J. Pharm. Res..

[132] Kipanga P.N., Liu M., Panda S.K., Mai A.H., Veryser C., van Puyvelde L., de Borggraeve W.M., van Dijck P., Matasyoh J., Luyten W. (2020). Biofilm inhibiting properties of compounds from the leaves of *Warburgia ugandensis* Sprague subsp ugandensis against Candida and staphylococcal biofilms. J. Ethnopharmacol..

[133] Nwogwugwu J.O., Batcho A.A. (2019). Antifungal potentials of some botanicals on *Sclerotium rolfsii* schum., the causal pathogen of damping-off of *Gmelina arborea* Roxb. in Ibadan, Southwestern Nigeria. J. Plant. Dis. Prot..

[134] Ourabah A., Atmani-Kilani D., Debbache-Benaida N., Kolesova O., Azib L., Yous F., Benloukil M., Botta B., Atmani D., Simonetti G. (2020). Anti-*Candida albicans* biofilm activity of extracts from two selected indigenous Algerian plants: *Clematis flammula* and *Fraxinus angustifolia*. J. Herb. Med..

[135] Rezgui M., Majdoub N., Mabrouk B., Baldisserotto A., Bino A., Kaab L.B., Manfredini S. (2020). Antioxidant and antifungal activities of marrubiin, extracts and essential oil from *Marrubium vulgare* L. against pathogenic dermatophyte strains. J. Mycol. Med..

[136] Salhi N., Mohammed Saghir S.A., Terzi V., Brahmi I., Ghedairi N., Bissati S. (2017). Antifungal activity of aqueous extracts of some dominant Algerian medicinal plants. BioMed Res. Int..

[137] Suurbaar J., Mosobil R., Donkor A.M. (2017). Antibacterial and antifungal activities and phytochemical profile of leaf extract from different extractants of *Ricinus communis* against selected pathogens. BMC Res. Notes.

[138] Umaru I.J., Badruddin F.A., Ha U. (2018). Antifungal potential of some medicinal plants on selected pathogenic fungi. MOJ Proteom. Bioinform..

[139] Maema L.P., Potgieter M., Masevhe N.A., Samie A. (2020). Antimicrobial activity of selected plants against fungal species isolated from South African AIDS patients and their antigonococcal activity. J. Complement. Integr. Med..

[140] Siegel R.L., Miller K.D., Jemal A. (2015). Cancer statistics. CA Cancer J. Clin..

[141] Bollyky T.J., Andridge C. (2015). Cancer Prevention and Treatment in Developing Countries: Recommendations for Action Cancer Control.

[142] Bray F., Ferlay J., Soerjomataram I., Siegel R.L., Torre L.A., Jemal A. (2018). Global cancer statistics: GLOBOCAN estimates of incidence and mortality worldwide for 36 cancers in 185 countries. CA Cancer J. Clin..

[143] Mans D.R., da Rocha A.B., Schwartsmann G. (2000). Anti-cancer drug discovery and development in Brazil: Targeted plant collection as a rational strategy to acquire candidate anti-cancer compounds. Oncolgist.

[144] Newman D.J. (2008). Natural products as leads to potential drugs: An old process or the new hope for drug discovery?. J. Med. Chem..

[145] Shamim G., Ranjan S.K., Pandey D.M., Sharma K.K., Ramani R. (2016). Lac dye as a potential anti-neoplastic agent. J. Cancer Res. Ther..

[146] Tiwari R., Latheef S.K., Ahmed I., Iqbal H., Bule M.H., Dhama K., Samad H.A., Karthik K., Alagawany M., El-Hack M.E. (2018). Herbal immunomodulators-a remedial panacea for designing and developing effective drugs and medicines: Current scenario and prospects. Curr. Drug Metab..

[147] Fridlender M., Kapulnik Y., Koltai H. (2015). Plant derived substances with anti-cancer activity: From folklore to practice. Front. Plant Sci..

[148] Catalano E., Maestri P., Cochis A., Azzimonti B., Varoni E., Fraschini F., Rimondini L., Megna S., Iriti M. (2014). Cytotoxic activity of plant extract on cancer cells. Ital. J. Anat. Embryol..

[149] Kumar R., Sharma M. (2018). Herbal nanomedicine interactions to enhance pharmacokinetics, pharmaco-dynamics, and therapeutic index for better bioavailability and biocompatibility of herbal formulations. J. Mater. NanoSci..

[150] Kumar G., Gupta R., Sharan S., Roy P., Pandey D.M. (2019). Anticancer activity of plant leaves extract collected from a tribal region of India. 3 Biotech.

[151] Kawashima K., Misawa H., Moriwaki Y. (2007). Ubiquitous expression of acetylcholine and its biological functions in life forms without nervous systems. Life Sci..

[152] Balasubramanian K., Padma P.R. (2013). Anticancer activity of *Zea mays* leaf extracts on oxidative stress-induced Hep2 Cells. J. Acupunct. Meridian Stud..

[153] Bauml J.M., Chokshi S., Schapira M.M. (2015). Do attitudes and beliefs regarding complementary and alternative medicine impact its use among patients with cancer? A cross-sectional survey. Cancer.

[154] Kondhare D., Lade H. (2017). Phytochemical profile, aldose reductase inhibitory, and antioxidant activities of Indian traditional medicinal *Coccinia grandis* (L.) fruit extract. 3 Biotech.

[155] Hawk M.A., McCallister C., Schafer Z.T. (2016). Antioxidant activity during tumour progression: A necessity for the survival of cancer cells?. Cancers.

[156] Lobo V., Patil A., Phatak A., Chandra N. (2010). Free radicals, antioxidants and functional foods: Impact on human health. Pharmacogn. Rev..

[157] Kaurinovic B., Vastag D. (2019). Flavonoids and phenolic acids as potential natural antioxidants. Antioxidants.

[158] Adebiyi O.E., Olayemi F.O., Ning-Hua T., Guang-Zhi Z. (2017). In vitro antioxidant activity, total phenolic and flavonoid contents of ethanol extract of stem and leaf of *Grewia carpinifolia*. Beni-Seuf Univ. J. Basic Appl. Sci..

[159] Rahman M.A., Bjada S., Akhtar J. (2016). Evaluation of anticancer activity of *Cordia dichotoma* leaves against a human prostate carcinoma cell line, PC3. J. Tradit. Complement. Med..

[160] Efferth T., Koch E. (2011). Complex interactions between phytochemicals. The multi-target therapeutic concept of phytotherapy. Curr. Drug Targets.

[161] Wöll S., Kim S., Efferth T. (2013). Animal plant warfare and secondary metabolite evolution. Nat. Prod. Bioprospect..

[162] Bouyahya A., Bakri Y., Et-Touys A., Assemian I.C.C., Abrini J., Dakka N. (2018). In vitro antiproliferative activity of selected medicinal plants from the North-West of Morocco on several cancer cell lines. Eur. J. Integr. Med..

[163] Alabi M.A., Muthusamy A., Kabekkodu S.P., Adebawo O.O., Satyamoorthy K. (2020). Anticancer properties of recipes derived from Nigeria and African medicinal plants on breast cancer cells in vitro. Sci. Afr..

[164] Tonisi S., Okaiyeto K., Mabinya L.V., Okoh A.I. (2020). Evaluation of bioactive compounds, free radical scavenging, and anticancer activities of bulb extracts of *Boophone disticha* from Eastern Cape Province, South Africa. Saudi J. Biol. Sci..

[165] Onyancha J.M., Gikonyo N.K., Wachira S.W., Mwitari P.G., Gicheru M.M. (2018). Anticancer activities and safety evaluation of selected Kenyan plant extracts against breast cancer cell lines. J. Pharmacogn. Phytother..

[166] Akindele A.J., Wani Z., Mahajan G., Sharma S., Aigbe F.R., Satti N., Adeyemi O.O., Mondhe D.M. (2015). Anticancer activity of *Aristolochia ringens* Vahl. (Aristolochiaceae). J. Tradit. Complement. Med..

[167] Saeed M.E., Abdelgadir H., Sugimoto Y., Khalid H.E., Efferth T. (2015). Cytotoxicity of 35 medicinal plants from Sudan towards sensitive and multidrug-resistant cancer cells. J. Ethnopharmacol..

[168] Mongalo N.I., Soyingbe O.S., Makhafola T.J. (2019). Antimicrobial, cytotoxicity, anticancer and antioxidant activities of *Jatropha zeyheri* Sond. roots (Euphorbiaceae). Asian Pac. J. Trop. Biomed..

[169] Najjaa H., Abdelkarim B.A., Doria E., Boubakri A., Trabelsi N., Falleh H., Tlili H., Neffati M. (2020). Phenolic composition of some Tunisian medicinal plants associated with anti-proliferative effect on human breast cancer MCF-7 cells. EuroBiotech. J..

[170] Elnour M.A., Penech F., Mesaik M. (2017). Four selected Sudanese medicinal plants induce anticancer and cytotoxic effects in prostate cancer cell line. Clin. Med. Biochem..

[171] Tlili H., Hanen N., Ben A.A., Neffati M., Boubakri A., Buonocore D., Dossena M., Verri M., Doria E. (2019). Biochemical profile and in vitro biological activities of extracts from seven folk medicinal plants growing wild in southern Tunisia. PLoS ONE.

[172] Twilley D., Langhansová L., Palaniswamy D., Lall N. (2017). Evaluation of traditionally used medicinal plants for anticancer, antioxidant, anti-inflammatory and anti-viral (HPV-1) activity. S. Afr. J. Bot..

[173] Taylor P.G., Cesari I.M., Arsenak M., Ballen D., Abad M.J., Fernández A., Milano B., Ruiz M.C., Williams B., Michelangeli F. (2006). Evaluation of Venezuelan medicinal plant extracts for antitumor and antiprotease activities. Pharm. Biol..

[174] Acheampong F., Larbie C., Appiah-Opong R., Arthur F.K., Tuffour I. (2015). In vitro antioxidant and anticancer properties of hydroethanolic extracts and fractions of *Ageratum conyzoides*. Eur. J. Med. Plants.

[175] Tamokou de Dieu Chouna J., Fischer-Fodor J.R., Chereches G., Barbos O., Damian G., Benedec D., Duma M., Efouet A.P.N., Wabo H.K., Kuiate J.R. (2013). Anticancer and antimicrobial activities of some antioxidant-rich Cameroonian medicinal plants. PLoS ONE.

[176] Tuasha N., Seifu D., Gadisa E., Petros B., Oredsson S. (2019). Cytotoxicity of selected Ethiopian medicinal plants used in traditional breast cancer treatment against breast-derived cell lines. J. Med. Plants Res..

[177] Mukanganyama S., Dumbura S.C., Mampuru L. (2012). Anti-proliferative effects of plant extracts from Zimbabwean medicinal plants against human leukaemic cell lines. Afr. J. Plant. Sci. Biotechnol..

[178] Ndhlala A.R., Amoo S.O., Ncube B., Moyo M., Nair J.J., van Staden J., Kuete V. (2013). Antibacterial, antifungal, and antiviral activities of african medicinal plants. Medicinal Plant Research in Africa.

[179] Wagner E.K., Hewlett M.J. (1999). Basic Virology.

[180] de Clercq E., Field H.J. (2006). Antiviral prodrugs–the development of successful prodrug strategies for antiviral chemotherapy. Br. J. Pharmacol..

[181] Jaime M.F.V., Redko F., Muschietti L.V., Campos R.H., Martino V.S., Cavallaro L.V. (2013). In vitro antiviral activity of plant extracts from Asteraceae medicinal plants. Virol. J..

[182] Henkin J.M., Sydara K., Xayvue M., Souliya O., Kinghorn A.D., Burdette J.E., Chen W.L., Elkington B.G., Soejarto D.D. (2017). Revisiting the linkage between ethnomedical use and development of new medicines: A novel plant collection strategy towards the discovery of anticancer agents. J. Med. Plant Res..

[183] Mehrbod P., Abdalla M.A., Njoya E.M., Ahmed A.S., Fotouhi F., Farahmand B., Gado D.A., Tabatabaian M., Fasanmi O.G., Eloff J.N. (2018). South African medicinal plant extracts active against influenza A virus. BMC Complement. Altern. Med..

[184] Todorov D., Shishkova K., Dragolova D., Hinkov A., Kapchina-Toteva V., Shishkov S. (2015). Antiviral activity of medicinal plant Nepeta nuda. Biotechnol. Biotechnol. Equip..

[185] Ogbole O.O., Akinleye T.E., Segun P.A., Faleye T.C., Adeniji A.J. (2018). In vitro antiviral activity of twenty-seven medicinal plant extracts from Southwest Nigeria against three serotypes of echoviruses. Virol. J..

[186] Galani B.R., Sahuc M.E., Njayou F.N., Deloison G., Mkounga P., Feudjou W.F., Brodin P., Rouillé Y., Nkengfack A.E., Moundipa P.F. (2015). Plant extracts from Cameroonian medicinal plants strongly inhibit hepatitis C virus infection in vitro. Front. Microbiol..

[187] Mthethwa N.S., Oyedeji B.A., Obi L.C., Aiyegoro O.A. (2014). Anti-staphylococcal, anti-HIV and cytotoxicity studies of four South African medicinal plants and isolation of bioactive compounds from *Cassine transvaalensis* (Burtt. Davy) codd. BMC Complement. Altern. Med..

[188] Radol A.O., Kiptoo M., Makokha A.O., Tolo F.M. (2019). Determination of antioxidant value and chemical groups in selected medicinal plants used for conditions associated with herpes simplex and herpes Zoster Infections in Kakamega County, Kenya. Asian J. Biol..

[189] Tolo F.M., Rukunga G.M., Muli F.W., Njagi E.N., Njue W., Kumon K., Mungai G.M., Muthaura C.N., Muli J.M., Keter L.K. (2006). Anti-viral activity of the extracts of a Kenyan medicinal plant *Carissa edulis* against herpes simplex virus. J. Ethnopharmacol..

[190] Nwodo U.U., Ngene A.A., Iroegbu C.U., Onyedikachi O.A.L., Chigor V.N., Okoh A.I. (2011). In vivo evaluation of the antiviral activity of *Cajanus cajan* on measles virus. Arch. Virol..

[191] Ojo O.O., Oluyege J.O., Famurewa O. (2009). Antiviral properties of two Nigerian plants. Afr. J. Plant Sci..

[192] Bessong P.O., Obi C.L., Andre´ola M.L., Rojas L.B., Pouyse´gu L., Igumbor E., Meyer J.M., Quideau S., Litvak S. (2005). Evaluation of selected South African medicinal plants for inhibitory properties against immunodeficiency virus type 1 reverse transcriptase and integrase. J. Ethnopharmacol..

[193] Klos M., van de Venter M., Milne P.J., Traore H.N., Meyer D., Oosthuizen V. (2009). In vitro anti-HIV activity of five selected South African medicinal plant extracts. J. Ethnopharmacol..

[194] Sunday O.A., Munir A.B., Akeeb O.O., Bolanle A.A., Badaru S.O. (2010). Antiviral effect of *Hibiscus sabdariffa* and *Celosia argentea* on measles virus. Afr. J. Microbiol. Res..

